# Draft Genome Sequence of Dermacoccus nishinomiyaensis TSA37, Isolated from Wood Ash

**DOI:** 10.1128/MRA.01370-19

**Published:** 2019-12-12

**Authors:** Alexander N. Williams, Kyle S. MacLea

**Affiliations:** a Biotechnology Program, University of New Hampshire, Manchester, New Hampshire, USA; b Biology Program, University of New Hampshire, Manchester, New Hampshire, USA; c Department of Life Sciences, University of New Hampshire, Manchester, New Hampshire, USA; University of Arizona

## Abstract

Dermacoccus nishinomiyaensis is a common bacterial resident of the human skin microbiome, among other environments. *D. nishinomiyaensis* strain TSA37 was isolated from the ash pan of a residential wood pellet stove. A genome assembly of 3,130,592 bp was generated, with an *N*_50_ value of 197,547 bp and a calculated G+C content of 69.01%.

## ANNOUNCEMENT

Dermacoccus nishinomiyaensis is a species of aerobic Gram-positive cocci of the family *Dermacoccaceae* (1, 2). It is a known skin commensal (3) and is largely nonpathogenic to humans, though recent studies have implicated *D. nishinomiyaensis* in peritoneal dialysis-related peritonitis (4), catheter-related bacteremia (5), and polymicrobial infections of the skin (6) and urinary tract (7, 8). Beyond the human skin microbiome, *D. nishinomiyaensis* has also been isolated from an indoor track facility (9), cured meat (10), well water (11), and the gut of adult *Sarcophaga* flesh flies (12). Given a growing awareness of *D. nishinomiyaensis* beyond the human skin in other areas of the human-built environment, comparisons of skin commensal strains with strains found in other human-adjacent spaces may shed light on conditions under which the strains may exhibit pathogenicity.

*D. nishinomiyaensis* strain TSA37 was isolated from the ash pan of a residential wood pellet stove. A 30-ml sample of wood ash was collected, and a small portion was spread using a sterile cotton-tipped applicator onto a tryptic soy agar (TSA) plate and incubated at 37°C for 48 hours. After it was subcultured on a TSA plate formulated with 50.0 mg/liter cycloheximide for 24 hours, a small circular yellow colony (Gram-positive cocci) was isolated. Genomic DNA was purified from 5 ml inoculated tryptic soy broth (grown at 37°C for 24 hours) using the QIAamp DNA minikit (Qiagen, Valencia, CA, USA). The purified genomic DNA was fragmented and tagged with sequence adapters using the HyperPlus kit v.3.16 (catalog number KR1145; KAPA, Wilmington, MA, USA) and then sequenced with an Illumina HiSeq 2500 instrument at the University of New Hampshire (UNH) Hubbard Center for Genome Studies (Durham, NH, USA). The resulting 250-bp paired-end reads were bioinformatically paired and trimmed using Trimmomatic v.0.38 (with the following settings: paired-end mode with a window size of 4, quality requirement of 15, and minimum read length of 36), and 1,927,890 trimmed reads covering 3,188,889 bp were then assembled into a draft genome sequence using SPAdes v.3.13.0 with default settings (13, 14) and assessed for quality measures using QUAST v.5.0.2 (15). Contaminants and contigs of <500 bp were removed, and genes and features on the remaining contigs were identified and annotated using the NCBI Prokaryotic Genome Annotation Pipeline (PGAP) v.4.8 (16).

Assignment of the strain to the species *D. nishinomiyaensis* was verified using BLAST (17), average nucleotide identity (98.05% using EzBioCloud) (18), and the NCBI SRA Taxonomy Analysis Tool (STAT) (not yet published but available on the NCBI Sequence Read Archive “Analysis” tab). *D. nishinomiyaensis* strain TSA37 was found to have a complete genome size of 3,130,592 bp across 37 contigs, with an average sequence coverage of 154×, an *N*_50_ value of 197,547 bp, and a G+C content of 69.01%. A total of 2,864 genes, 2,801 coding sequences, 93 pseudogenes, and 3 noncoding RNAs (ncRNAs) were identified using PGAP analysis. One CRISPR array was also detected using PGAP analysis. A BLASTn Megablast search of the 29-bp repeat sequence for the array returned *Pseudomonas* phage JG054 as the best hit, with marginal scoring (E value, 0.70; query coverage, 62%; identity, 100%).

### Data availability.

This Dermacoccus nishinomiyaensis whole-genome shotgun project has been deposited in DDBJ/ENA/GenBank under the accession number SUPQ00000000. The version described in this paper is the first version, SUPQ01000000. The raw Illumina data from BioProject PRJNA534294 were submitted to the NCBI Sequence Read Archive (SRA) under experiment accession number SRX6871076.
